# Bayes-optimal estimation of overlap between populations of fixed size

**DOI:** 10.1371/journal.pcbi.1006898

**Published:** 2019-03-29

**Authors:** Daniel B. Larremore

**Affiliations:** 1 Department of Computer Science, University of Colorado Boulder, Boulder, Colorado, United States of America; 2 BioFrontiers Institute, University of Colorado Boulder, Boulder, Colorado, United States of America; University of Chicago, UNITED STATES

## Abstract

Measuring the overlap between two populations is, in principle, straightforward. Upon fully sampling both populations, the number of shared objects—species, taxonomical units, or gene variants, depending on the context—can be directly counted. In practice, however, only a fraction of each population’s objects are likely to be sampled due to stochastic data collection or sequencing techniques. Although methods exists for quantifying population overlap under subsampled conditions, their bias is well documented and the uncertainty of their estimates cannot be quantified. Here we derive and validate a method to rigorously estimate the population overlap from incomplete samples when the total number of objects, species, or genes in each population is known, a special case of the more general *β*-diversity problem that is particularly relevant in the ecology and genomic epidemiology of malaria. By solving a Bayesian inference problem, this method takes into account the rates of subsampling and produces unbiased and Bayes-optimal estimates of overlap. In addition, it provides a natural framework for computing the uncertainty of its estimates, and can be used prospectively in study planning by quantifying the tradeoff between sampling effort and uncertainty.

## Author summary

Understanding when two populations are composed of similar species is important for ecologists, epidemiologists, and population geneticists, and in principle it is easy: just sample the two populations, compare the sets of species identified in each, and count how many appear in both populations. In practice, however, this is difficult because sampling methods typically produce only a random subset of the total population, leaving current population overlap estimates biased. Knowing only the number of shared members between two of these partial population samples, this paper shows how we can nevertheless estimate the true overlap between the full populations, when those full populations’ sizes are known. Using Bayesian statistics, we can also compute credible intervals to produce error bars. We show that using this unbiased approach has a dramatic impact on the conclusions one might draw from previously published studies in the malaria literature, which used simple but biased methods. Because the method in this paper quantifies the tradeoff between sampling effort and uncertainty, we also show how to compute the number of samples required to ensure high-confidence results, which may be useful for planning future studies or budgeting lab reagents and time.

This is a *PLoS Computational Biology* Methods paper.

## Introduction

Quantifying the similarity between two populations, environments, or ecosystems, based on their constituent members or species, is a fundamental problem in ecology. Some methods quantify this pairwise similarity, often called *β*-diversity [1], based on only the presence or absence of species [2], while other methods take into account species abundance as well [3]. Still other methods, more common in microbial ecology, make use of genetic sequence data, measuring similarities through phylogenetic relationships [4–6]. In practical applications of all three types of methods, the populations being compared are almost always undersampled, meaning that estimators which are principled in the context of perfect sampling show substantial bias in practice [7].

Consider, as an example of estimator bias, the oldest of pairwise similarity measures, which have roots in botany with Jaccard, Dice, and Sørenson. Their 1901 [8] and 1940s [9, 10] publications introduced the eponymous Jaccard index and Sørenson-Dice coefficient. Both are simple ratios involving the number of distinct species observed in each population, *n*_*a*_ and *n*_*b*_, and the number of species shared by both populations, *n*_*ab*_, so that each measure quantifies overlap as a fraction,

$$\begin{array}{c} \overset{˚}{J}=\frac{n_{ab}}{n_{a}+n_{b}-n_{ab}}\;,\;\overset{˚}{S}=\frac{n_{ab}}{\frac{1}{2}\left(n_{a}+n_{b}\right)}\;. \end{array}$$ (1)

Intuitively, when the two populations are identical, both $\overset{˚}{J}$ and $\overset{˚}{S}$ are one, and when two populations are entirely distinct, both are zero. However, imagine two populations of 10 species each in which 5 species are found in both populations. With perfect sampling, $\overset{˚}{J}=\frac{1}{3}$ and $\overset{˚}{S}=\frac{1}{2}$, but when only 9 of 10 species are drawn from each population, these indices, computed with empirically observed values, average $\text{E}[\overset{˚}{J}]=0.29$ and $\text{E}[\overset{˚}{S}]=0.45$, representing relative biases of −12% and −10% respectively. These biases, which are well documented [7], become worse as sampling rates fall.

Bias is not unique to the Jaccard and Sørenson-Dice coefficients, but instead affects all algebraic combinations of *n*_*a*_, *n*_*b*_, and *n*_*ab*_, of which over 20 have been proposed [2]. This is due to the fact that observed values of *n*_*a*_, *n*_*b*_, and *n*_*ab*_ are realizations of random variables, and in particular, *n*_*ab*_ necessarily shows a nonlinear dependence on *n*_*a*_ and *n*_*b*_. As a consequence, guides to navigating the multiple measures of *β* diversity—including both presence/absence and abundance measures—emphasize matching of estimators’ principles and the scientific questions they are meant to answer [11], but do not address underlying bias, variation, or uncertainty itself.

Progress toward unbiased estimators has been made, however. If estimators account for not just the presence or absence of species, but their abundance as well, they can be adjusted based on the effects of both observed and *unobserved* species. Chao et al. took a probabilistic view of such adjustments based on the underlying sampling process, resulting in modified Jaccard and Sørenson-Dice coefficients with substantially reduced bias [7]. Other successful approaches directly model the sampling process itself. For instance, Kery and Royle introduced a hierarchical Bayes approach to species-richness estimation by posing a spatial sampling process and using it to improve richness estimates [12]. Importantly, this Bayesian approach allowed them to quantify uncertainty in their estimates via credible intervals. Outside of ecology entirely, the estimation of overlap between sets arises in large-data scenarios, e.g. when comparing two individuals’ sets of interests or friends on Facebook using streaming algorithms in distributed settings [13]. These approaches show that better estimates are possible when the presence of uncertainty due to stochastic sampling is addressed directly, even when much about the underlying populations is unknown.

Here, we solve a special case of the more general pairwise similarity problem described above—one which is particularly relevant to the genomic epidemiology and disease ecology of *Plasmodium falciparum*, the most virulent of the human malaria parasites. Rather than comparing two ecosystems based on their shared species, we consider the problem of comparing two genomic repertoires based on their shared gene variants. Mathematically, the problems are similar but with one important difference: when estimating genomic repertoire overlap, the total number of variants per genome is known. This additional specification opens the door to unbiased and Bayes-optimal estimation of true repertoire overlap, given a single noisy measurement of *n*_*a*_, *n*_*b*_, and *n*_*ab*_, while also quantifying the increased uncertainty inherent in decreased sampling.

### The *P*. *falciparum* repertoire overlap problem

Of the diverse multigene families of *P*. *falciparum*, the *var* family is the most heavily studied because of its direct links to both malaria’s duration of infection and its virulence [14–17]. Each parasite genome contains a repertoire of ∼ 60 hypervariable and mutually distinct *var* genes, but repertoires differ between parasites, evolving rapidly through recombination and reassortment. Recent studies of *P*. *falciparum* epidemiology and evolution have generated insights by comparing of the sets of genomic *var* repertoires between parasites [18–23]. Indeed, since *var* repertoires are, themselves, under selection, theory suggests that if a human population has been exposed to particular *var* genes, then repertoires containing those *var* genes will have a lower fitness than repertoires that are entirely unrecognized by local hosts, shaping the *var* population structure [21, 22, 24, 25]. Methods by which we estimate the extent to which *var* repertoires overlap are therefore important, particularly as studies of the population genetics and genetic epidemiology of malaria’s antigens become more sophisticated and data rich. However, as with estimates of *β*-diversity in ecology, traditional estimates of overlap between *var* repertoires also suffer bias due to subsampling.

**Fig 1:**
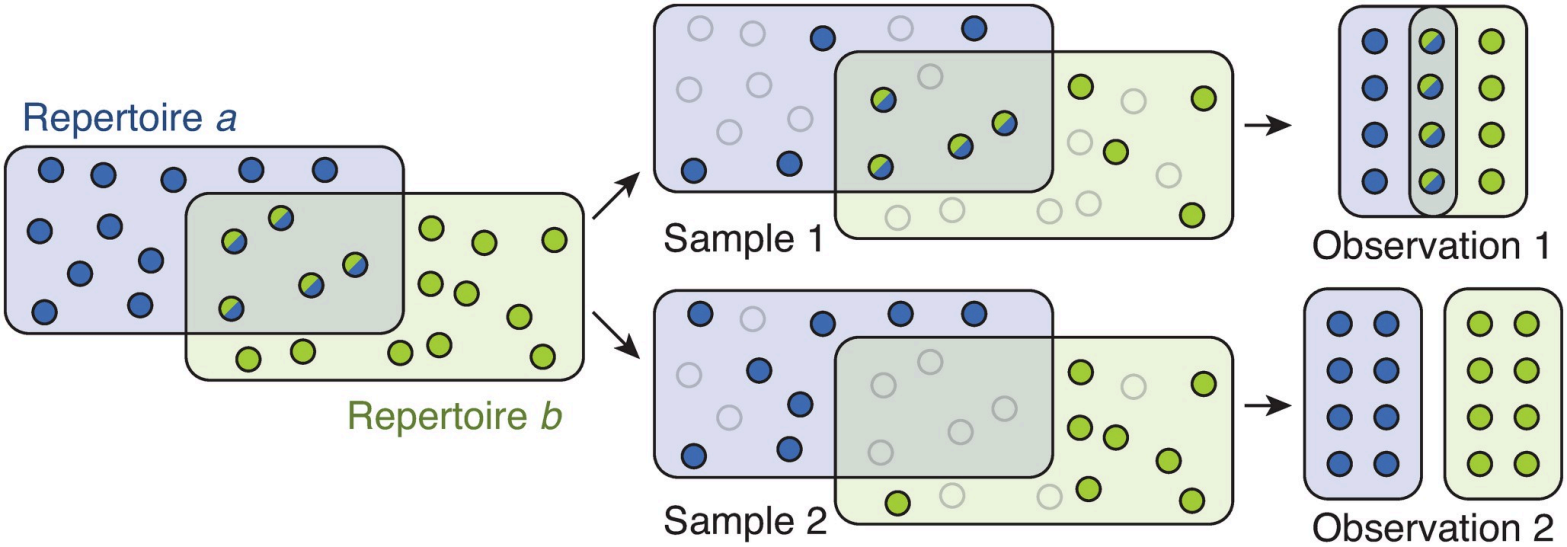
Stochastic sampling leads to variation in observed overlap. The members of two hypothetical populations are represented by blue and green circles, respectively. Each population has 16 members, and *s* = 5 are shared members of both populations. In two independent sampling experiments, shown in top and bottom rows, *n*_*a*_ = *n*_*b*_ = 8 members are sampled at random from each population (dark circles) while the other 8 members are not sampled (transparent circles). Observation of the first experiment finds an overlap of *n*_*ab*_ = 4, while observation of the second finds *n*_*ab*_ = 0.

Due to the massive diversity and recombinant structure of *var* genes, researchers are restricted to using degenerate PCR primers targeting a small “tag” sequence within a particular *var* domain called DBL*α* [26]. Due to their experimental accessibility, DBL*α* tags have been widely used to study the structure and function of *var* genes [17, 18, 21, 26–30]. Still, these PCR techniques generate a random sample of 60 or fewer unique tag sequences from each parasite. This means that experimental measurements of repertoire overlap are performed using stochastic subsamples whose *empirical* overlap may fluctuate from experiment to experiment (Fig 1), motivating the three questions answered by this paper: First, how can we estimate the true overlap between repertoires when we can only measure the overlap between samples from repertoires? Second, how can we quantify the uncertainty around our repertoire overlap estimates? Third, what are the implications of uncertainty for the design and budgeting of *var* repertoire studies?

In the malaria literature, repertoire overlap is most commonly computed using the Sørenson-Dice coefficient where it is often called *pairwise type sharing* [18]. When PCR methods have produced *n*_*a*_ and *n*_*b*_ tags from parasites *a* and *b*, respectively, and when a sequence-level comparison has found *n*_*ab*_ tags are shared by both repertoires then repertoire similarity is computed using the coefficient $\overset{˚}{S}$ in Eq (1). When *n*_*a*_ and *n*_*b*_ are nearly 60, the performance of $\overset{˚}{S}$ is excellent. For instance, when two parasites are completely different, *n*_*ab*_ = 0, so $\overset{˚}{S}=0$; when two parasites are identical, and both repertoires have been fully sampled, *n*_*ab*_ = *n*_*a*_ = *n*_*b*_, so $\overset{˚}{S}=1$. However, when *n*_*a*_ or *n*_*b*_ is smaller (as is overwhelmingly the case in existing studies [18–23]) $\overset{˚}{S}$ is conservative and systematically underestimates the true overlap between repertoires [7].

### Organization

In this manuscript, I introduce a method that estimates repertoire overlap using Bayesian inference. By modeling the stochastic process by which repertoires are sampled, I show that this method produces unbiased *a posteriori* estimates of true repertoire overlap. I then show how the Bayesian framework can be used to estimate uncertainty and produce error bars which represent credible intervals, a Bayesian analog of confidence intervals. These methods are then used to reevaluate past results which used the Sørenson-Dice coefficient $\overset{˚}{S}$. Finally, in the case of *P*. *falciparum*, since each successful PCR amplification randomly samples just one of 60 available tags, I extend the Bayesian approach to compute the tradeoff between increasing sampling and decreasing the uncertainty of overlap estimates. These calculations allow the cost of sampling to be weighed against scientific confidence, illustrating the use of this statistical framework for planning and budgeting experiments. Open-source code and a web tool are freely available (see Acknowledgements).

## Methods

Suppose that there are two *P*. *falciparum* parasites, each with a repertoire of 60 *var* types. Our goal is to estimate the true repertoire overlap *s* (were we to fully sample each parasite) from the knowledge that *n*_*a*_ samples from parasite *a* and *n*_*b*_ samples from parasite *b* share *n*_*ab*_ types. Due to the fact that the underlying sampling process is stochastic (Fig 1), our secondary goal is to quantify the uncertainty in the method’s estimates. Both goals can be met by writing down the process that creates the data in the first place. Therefore, in what follows, we will at first assume that the true overlap *s* is fixed, model the process of generating data via stochastic sampling, and use that model to compute a likelihood. We will then use Bayes’ Rule to compute the posterior probability for each value of *s*, given the evidence in the data and the likelihood computed in the first step.

Consider the following sampling process, written in the slightly more rigid and generic language of a probability textbook. Suppose that there are *s* special objects among a total of *N* objects. We draw *n* objects uniformly at random without replacement. The number of special objects chosen during this sampling procedure will be distributed according to a hypergeometric distribution, which we write as H(s,N,n).

First, with this definition in mind, consider drawing *n*_*a*_ *var* genes from parasite *a*’s 60 total. Of the 60 total, suppose that exactly *s* are considered special because they are also shared by parasite *b*. The number of shared sequences that are captured by sequencing parasite *a* will be a random variable $S_{a}=H\left(s,60,n_{a}\right)$. Depending on the luck of the draw, this number could be as small as zero, or as high as *s* or *n*_*a*_ (whichever is smaller).

Now consider drawing *n*_*b*_ *var* genes from parasite *b*’s 60 total, in which exactly *s*_*a*_ are special because they are shared by both parasites *and* were actually drawn during the sequencing of parasite *a*. This process is identical in construction to the process for sampling parasite *a*, but with *s*_*a*_ special sequences instead of *s*, and so the number of shared sequences that are captured after sequencing both parasites will be $H(s_{a},60,n_{b})$. Substituting the random variable *S*_*a*_ for a fixed value *s*_*a*_, which we derived in the paragraph above, yields a hypergeometric inside a hypergeometric, which means that the probability of a particular number of shared sequences in the samples *n*_*ab*_ is given by these sequential (or nested) hypergeometric distributions,

$$\begin{array}{c} P\left(n_{ab}|n_{a},n_{b},s\right)∼H(H\left(s,60,n_{a}\right),60,n_{b})\;. \end{array}$$ (2)

Reassuringly, one can switch the order in which the imagined sampling took place, first sequencing parasite *b* and then sequencing parasite *a*, or sequencing them both at once, and show that these are mathematically equivalent.

In practice, we want to go the other direction, and estimate *s* from our empirical measurements of *n*_*a*_, *n*_*b*_, and *n*_*ab*_. Since the distributions above allow us to compute the likelihood of empirical observations, given *s*, we use Bayes’ rule to formulate the posterior distribution for *s*,

$$\begin{array}{c} P\left(s|n_{a},n_{b},n_{ab}\right)=\frac{P\left(n_{ab}|n_{a},n_{b},s\right)P\left(s\right)}{P(n_{ab})}\;, \end{array}$$ (3)

where *P*(*s*) is the prior distribution for overlap. In practice, we generally wish to remain agnostic about the level of overlap *s* and therefore we consider an uninformative prior *P*(*s*) ∼ unif[0, 60], i.e. $P\left(s\right)=\frac{1}{61}$. Using the law of total probability to rewrite the denominator, and canceling the factors of $\frac{1}{61}$, we get

$$\begin{array}{c} P\left(s|n_{a},n_{b},n_{ab}\right)=\frac{P(n_{ab}|n_{a},n_{b},s)}{\sum_{s^{\prime }=0}^{60}P\left(n_{ab}|n_{a},n_{b},s^{\prime }\right)}\;. \end{array}$$ (4)

Each term on the right hand side of Eq (4) can now be computed directly from the nested hypergeometric distributions in Eq (2) as follows. To generate a specific empirical overlap *n*_*ab*_, two things must have happened in succession and independently of each other: first, *s*_*a*_ of the original *s* shared sequences must have been sampled; and second, *n*_*ab*_ of the intermediate *s*_*a*_ shared sequences must then have been sampled. We therefore multiply these two hypergeometric probabilities. However, because this sequential process may occur for any value of the intermediate variable *s*_*a*_, we sum over all possible values of *s*_*a*_,

$$\begin{array}{c} P\left(n_{ab}|n_{a},n_{b},s\right)=\sum_{s_{a}=0}^{60}P\left(n_{ab}|n_{b},s_{a}\right)P\left(s_{a}|n_{a},s\right)\;. \end{array}$$ (5)

Thus, computing the posterior probability that the true overlap was *s*, given the empirical overlap between samples, is given by substituting Eq (5) into Eq (4), yielding

$$\begin{array}{c} P\left(s|n_{a},n_{b},n_{ab}\right)=\frac{\sum_{s_{a}=0}^{60}P\left(n_{ab}|n_{b},s_{a}\right)P\left(s_{a}|n_{a},s\right)}{\sum_{s^{\prime }=0}^{60}\sum_{s_{a}=0}^{60}P\left(n_{ab}|n_{b},s_{a}\right)P\left(s_{a}|n_{a},s^{\prime }\right)}\;. \end{array}$$ (6)

**Fig 2:**
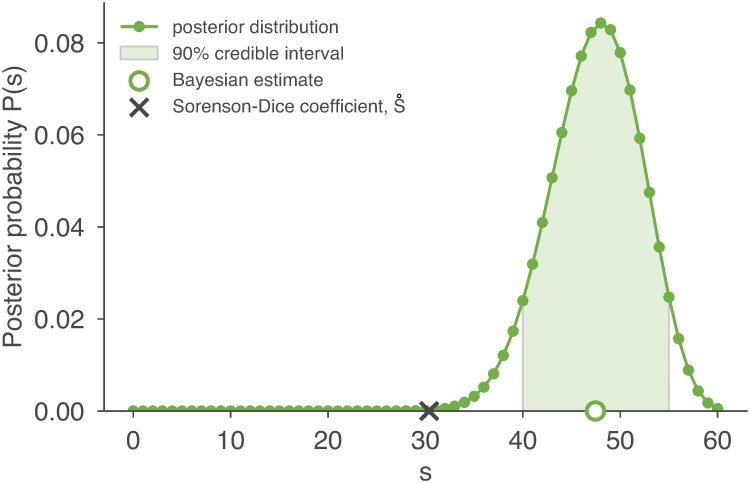
Inference and uncertainty using the posterior. The posterior distribution over *s* is plotted for the realistic scenario of *n*_*a*_ = 47, *n*_*b*_ = 32, and *n*_*ab*_ = 20 [line; Eq (6)]. The posterior mean provides our estimate of the true overlap $\hat{s}$ [open circle; Eq (7)], and the interval accounting for at least 90% of the area under the posterior curve provides an equal-tailed 90% credible interval [shading; Eq (8)]. The $\overset{˚}{S}$ estimate is shown for comparison [black cross; Eq (1)], and is typically less than or equal to $\hat{s}$.

The term *P*(*s* ∣ *n*_*a*_, *n*_*b*_, *n*_*ab*_) is a posterior distribution over *s*, meaning that it tells us the probability for each value of *s*, given the evidence provided by the actual data. While this equation appears notation-heavy, its inference requires only calls to the hypergeometric probability distribution. To illustrate this graphically, the posterior distribution is plotted for *n*_*a*_ = 47, *n*_*b*_ = 32, and *n*_*ab*_ = 20 in Fig 2.

The posterior distribution can now be used (i) to estimate the true value of *s*, and (ii) to quantify the uncertainty of that estimate. First, our estimate for the true value of *s*, which we call $\hat{s}$, is the expected value of the posterior,

$$\begin{array}{c} \hat{s}=\sum_{s=0}^{60}s\;P\left(s|n_{a},n_{b},n_{ab}\right). \end{array}$$ (7)

This value is typically (in 99.85% of all possible cases) larger than the estimate provided by $\overset{˚}{S}$ (Fig 2).

The framework here is easily extended to repertoire sizes other than 60, generalizing to applications beyond *var* genes. Suppose that populations *a* and *b* have total sizes of *N*_*a*_ and *N*_*b*_, and without loss of generality, assume that *N*_*a*_ ≤ *N*_*b*_. The values *N*_*a*_ and *N*_*b*_ need only be substituted into Eqs (6) and (7), with $P\left(s\right)=N_{a+1}^{-1}$. This is done explicitly in Eqs. (1) and (2) in S1 Text, but not shown here for conciseness (see S1 Text).

The posterior distribution provides a convenient way to quantify the uncertainty associated with an estimate $\hat{s}$. Intuitively, if the posterior is sharply peaked around $\hat{s}$, then our confidence in $\hat{s}$ is high; if the posterior is broadly distributed then our confidence in $\hat{s}$ is low. Making use of the Bayesian construction once more, we compute a credible interval by finding the range of *s* values that account for 90% of the posterior probability (Fig 2). Due to the fact that the posterior distribution is a discrete distribution over only 61 values, it is possible (indeed, highly probable) that no interval will contain exactly 90% of the probability. Nevertheless, we define a conservative equal-tailed 90% credible interval [*s*_min_, *s*_max_] as the smallest index *s*_min_ and the largest index *s*_max_ for which

$$\begin{array}{c} \sum_{s=s_{\text{max}}}^{60}P\left(s|n_{a},n_{b},n_{ab}\right)\geq 0.05\\ \sum_{s=0}^{s_{\text{min}}}P\left(s|n_{a},n_{b},n_{ab}\right)\geq 0.05\;. \end{array}$$ (8)

## Results

### Estimator performance

We first demonstrate that the $\hat{s}$ computed in Eq (7) produces accurate estimates by simulating the sampling process with known *s* and evaluating our ability to accurately recover it. Specifically, for each simulation, we consider two *var* repertoires *a* and *b*, of 60 genes each, and specify a priori that they share exactly *s* sequences. We then choose the number of samples taken from each, *n*_*a*_ and *n*_*b*_ respectively, and draw from each repertoire uniformly at random, without replacement. These draws are compared to compute the number of empirically shared sequences *n*_*ab*_. Eq (7) is used to compute the Bayesian repertoire overlap (BRO) estimate $\hat{s}$, while Eq (1) is used to compute $\overset{˚}{S}$ using the same data. These estimates are then compared to the true value of *s* to evaluate accuracy. Varying the values of *s*, *n*_*a*_, and *n*_*b*_ allows us to quantify the performance of BRO and $\overset{˚}{S}$ in a variety of realistic sampling scenarios.

**Fig 3:**
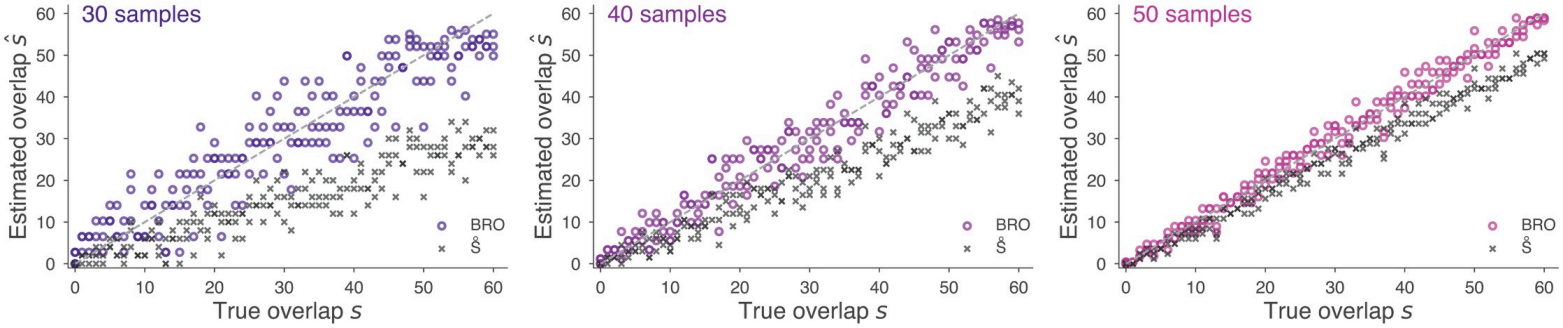
Bayesian repertoire overlap consistently estimates true overlap. Repertoires with true overlaps ranging from 0 to 60 were subsampled in simulations. As sampling rates increase from *n*_*a*_ = *n*_*b*_ = 30 (left) to 40 (middle) and to 50 (right), the estimates of BRO (colored circles) approach the true values (dotted lines) symmetrically. Estimates from $\overset{˚}{S}$ (crosses) approach the true values from below, systematically underestimating the true overlap. This bias is worse with lower sampling rates [7]. Similar results are found when *n*_*a*_ ≠ *n*_*b*_, and when the total repertoire sizes are different from each other (S1 Fig).

Fig 3 shows the results of this simulation for sampling rates of 30, 40, and 50 genes, with two independent simulations at each value of *s*. Intuitively, both BRO and $\overset{˚}{S}$ are more accurate when *n*_*a*_ and *n*_*b*_ are larger. However, the two methods’ behaviors are fundamentally different. When *n*_*a*_ and *n*_*b*_ are below 60, BRO provides estimates that are distributed around the true overlap, with variance decreasing as sampling rates increase. In contrast, $\overset{˚}{S}$ systematically underestimates the true overlap, while also showing decreasing variance as sampling rates increase [7]. For realistic sampling rates, BRO provides estimates centered at the true value, while $\overset{˚}{S}$ provides estimates centered below the true value. These general patterns hold even when *n*_*a*_ ≠ *n*_*b*_ or when total repertoire sizes are unequal (S1 Fig).

**Fig 4:**
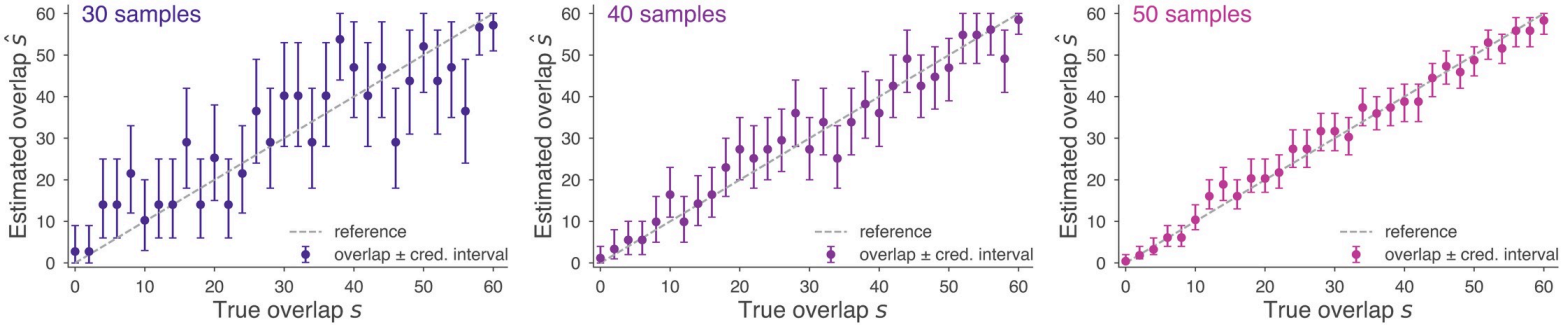
Credible intervals quantify uncertainty in overlap estimates. By using Eq (8), 90% credible intervals are show above as error bars around the point estimates $\hat{s}$ for varying true overlap *s*. As sampling rate increases from *n*_*a*_ = *n*_*b*_ = 30 (left) to 40 (middle) and to 50 (right), credible intervals shrink, indicating a reduction in uncertainty. In expectation, 90% of intervals cover the true overlap (dotted line).

Credible intervals, which visually show uncertainty in each estimate, can also be easily computed from the simulations described above. For each simulation, Eq (8) uses the posterior distribution over *s* to produce error bars around the point estimate $\hat{s}$, shown for sampling rates of 30, 40, and 50 in Fig 4. This illustrates the substantial reduction in uncertainty that comes with increased sampling rates. While all simulations shown here use *n*_*a*_ = *n*_*b*_, this is by no means required (S1 Fig), and in real data scenarios, is rare.

### Revisiting past results

We now show how the methods of this paper can be used in practical contexts by applying them to data from three published studies. In particular, this reanalysis highlights the impact of variation in sampling rates across studies, which creates variable bias in $\overset{˚}{S}$ calculations and produces misleading results. However, we also show that while using BRO in place of $\overset{˚}{S}$ sidesteps bias problems, the ability to quantify uncertainty with error bars highlights new problems. In short, the conclusions of previous studies may be worth reevaluating.

In 2007, Barry et al. introduced $\overset{˚}{S}$, which they referred to as *pairwise type sharing*, in an analysis of *var* data from Amele, Papua New Guinea [18]. In 2010, Albrecht et al. included Barry’s data in a broader analysis of *var* data from Ariquemes, Brazil [19] which also included sequences from a study by Bull et al. from Kilifi, Kenya for comparison [27]. Each one of these studies, individually, sequenced parasite isolates to a particular target depth, yet the studies varied in their coverage of repertoires. Since the bias of $\overset{˚}{S}$ depends on the number of samples (Fig 3; see also [7]), the variation of sampling rates across study populations means that different populations are biased downward by different amounts.

**Fig 5:**
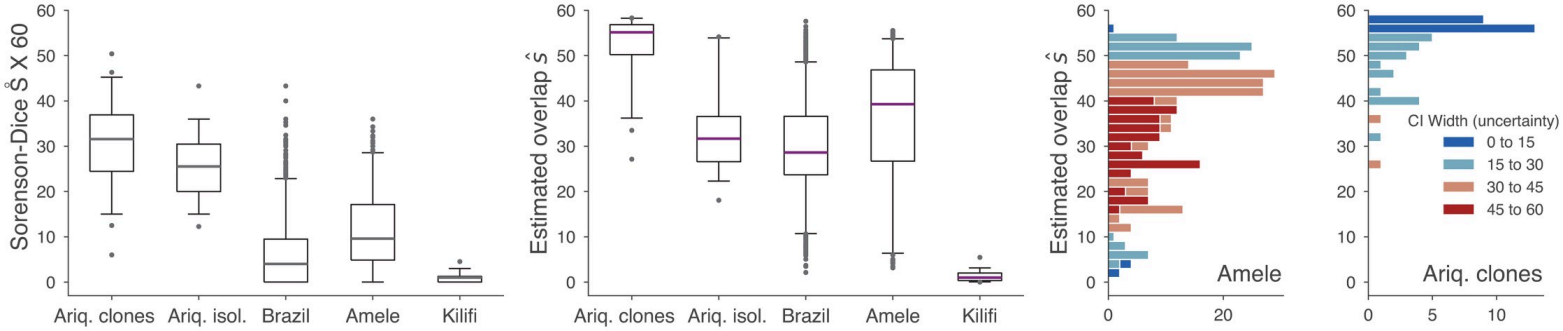
Reevaluation of published results. In 2010, Albrecht et al. compared *var* repertoires from 5 populations using pairwise type sharing (see Refs. [18, 19, 27] for original data details). (left) Reproduction of $\overset{˚}{S}$ analysis of [19], rescaled from [0, 1]→[0, 60]. (middle) Reanalysis using Bayesian repertoire overlap [Eq (7)]. For all boxplots, boxes span inner quartiles; center lines show medians; whiskers extend to 2.5 and 97.5 percentiles. (right) Histograms of Bayesian repertoire overlap distributions from Amele and Ariquemes clones (data identical to those in middle boxplots) colored by width of credible interval [Eq (8)], a measure of uncertainty. Differences in uncertainties are driven primarily by sampling rates: Amele samples average $\bar{n}\;=\;15.6$ sequences per parasite while Ariquemes clones average $\bar{n}\;=\;26.5$.

Albrecht et al. conveniently provide *var* type data from all three studies, from which we can rebuild their first figure which shows a $\overset{˚}{S}$ comparison of five populations (Fig 5; left). Overlaps between pairs of parasites can then be recomputed using BRO (Fig 5; middle). The conclusions drawn from these two figures differ substantially.

First, according to $\overset{˚}{S}$, identical clones from Ariquemes share only around 30 sequences with themselves, illustrating the downward bias produced by subsampling—clones ought to share all of their genes with their genetically identical siblings. Indeed, the reanalysis using BRO finds over 75% of overlap estimates to be greater than 50 (and over 50% over 55), far closer to what is expected.

Second, the inter-clone overlap and inter-isolate overlap distributions in Ariquemes appear to be similar and overlapping through the lens of $\overset{˚}{S}$. However, the recalculation using BRO shifts the clones’ distribution dramatically upward but leaves the isolates’ distribution more or less untouched. This is due to the dramatic difference in *var* coverage: the average number of sequences per clone is $\bar{n}\;=\;26.5$ while for isolates it is $\bar{n}\;=\;45.8$, meaning that relatively different amounts of bias are inherited from $\overset{˚}{S}$ (illustrated in simulations in Fig 3).

Finally, the distributions from Brazil ($\bar{n}\;=\;17.3$) and Amele ($\bar{n}\;=\;15.6$) also shift dramatically upward when the bias of $\overset{˚}{S}$ is removed (Fig 5; left, middle). However, this does not necessarily mean that they should be reinterpreted. For each pairwise comparison, Eq (8) allows us to compute the width of the credible interval, *s*_max_ − *s*_min_ + 1, quantifying our uncertainty in each estimate. Due to low average coverage, the uncertainty of estimates in the Amele dataset tends to be extremely large (Fig 5; right), with the majority of estimates showing an uncertainty greater than 30 sequences (50% overlap). For comparison, estimates from Ariquemes clones ($\bar{n}\;=\;26.5$) are also shown, whose dramatically lower uncertainty enables more confident conclusions to be drawn.

There are two main methodological findings that result from using rigorous and unbiased methods. First, the boxplots of Fig 5 clearly illustrate that sampling rates can have a dramatic impact on findings, reinforcing the simulation results of Fig 3. Second, uncertainty is an issue when $\bar{n}$ is too small, and datasets with low sampling rates may have such wide error bars that their estimates should not be trusted, as shown in the histograms of Fig 5, reinforcing the simulation results of Fig 4. Additional sequencing efforts come at a cost, however, and so in the next subsection we use the methods of this paper to quantify the tradeoff between increased sequencing efforts and decreased uncertainty.

### The cost of reduced uncertainty

In the previous section, the reanalysis of published results shows clearly that the number of samples per parasite has a dramatic impact on the uncertainty (and therefore the interpretability) of painstakingly collected parasite sequence data. Naturally, increasing the sampling rates, *n*_*a*_ and *n*_*b*_, decreases the uncertainty in $\hat{s}$, our estimate of *s* (Fig 4). However, additional samples cost time, effort, and money. Complicating matters, generating additional *var* sequences may or may not increase *n*_*a*_, since the previously sequenced *var* tags may be redundantly sequenced. Thus, there is a stochastic tradeoff between increased laboratory effort and decreased uncertainty about repertoire overlap, which we now calculate.

To obtain *var* tags, the DNA is PCR amplified using degenerate primers that are designed to universally capture all *var* genes with DBL*α* domains. This product is then cloned into a vector that allows single products to integrate, and these vectors are then transformed into bacteria and plated such that each colony contains one vector and one insert (see e.g. [21] for detailed methods, but see also [25] which uses a different pipeline based on next-generation sequencing). Therefore, among a large number of colonies, there are likely to be multiple colonies with the same *var* gene while some genes may not be covered by any colony. How many colonies should be separated and sequenced in order to get an accurate estimate of the repertoire overlap between two parasites? Put more formally, if we repeatedly perform an experiment in which we sequence *c* colonies each from two parasites and estimate their overlap $\hat{s}$, how much more accurate will $\hat{s}$ become if we increase *c*?

To answer this question, we split it into two parts. First, if we sequence *c* colonies, how many unique *var* genes *n* are we likely to have sampled? Second, what implications will this have for our repertoire overlap estimates, discussed in the previous section?

The first question can be answered by considering a process in which there are *k* = 60 distinct sequences in total and we draw *c* of them, one at a time, independently and with replacement. For a fixed *c*, we can compute the probability mass function for the number of distinct sequences by a straightforward recursion: At any point during the process of drawing sequences, if *n* distinct sequences have already been drawn, then the probability of drawing an already-discovered sequence is *n*/*k*, making the probability of drawing a new sequence 1 − *n*/*k*. Each draw is independent of the previous draws, so the incremental accumulation of distinct sequences can be written as a Markov chain with transition matrix *π* whose non-zero entries are

$$\begin{array}{c} \pi_{n\to n}=\frac{n}{k}\;\text{and}\;\pi_{n\to n+1}=1-\frac{n}{k}\;. \end{array}$$ (9)

Initially, zero sequences have been drawn (*c* = 0), making *n* = 0 with probability 1. For each additional sequence drawn, the probability distribution over the number of distinct sequences evolves according to the transition matrix *π*, so that after *c* draws the distribution over distinct sequences is given by the entries of the vector **x**,

$$\begin{array}{c} x=x_{0}^{T}\pi^{c}\;, \end{array}$$ (10)

where **x**_0_ is initial condition vector of zeros, except for the entry corresponding to the state *n* = 0, which equals one. This allows us to analytically compute the distribution of the number of unique *var* genes sampled by a PCR process with *c* colonies. In other words, we now have a map between laboratory efforts *c* and the distribution of actual unique *var* genes sampled, and we write this as *P*(*n* ∣ *c*). A variant of this problem was previously considered with the goal of computing the value of *c* that would cover at least 60% of each repertoire [31]. Although those calculations can be shown to produce incorrect estimates, Eq (10) can be used to solve that problem variant as well. More widely, this general problem has been charmingly named *the coupon collector’s problem* by statisticians.

The second question focuses on the implications of Eq (10), and specifically requires that we quantify how an increase in sequencing efforts *c* affects the noisy distribution of estimates $\hat{s}$. Intuitively, for low *c*, both *n*_*a*_ and *n*_*b*_ will tend to be small, leading to broad distributions of $\hat{s}$ around the correct value of *s*. Similarly, as *c* grows very large, we expect the distribution of $\hat{s}$ to concentrate on exactly *s*. This distribution, $P(\hat{s}|s,c)$, can be computed by integrating the distribution of estimates, conditioned on particular data, over the probability distribution of having produced those data, conditioned on *c* and *s*. Symbolically, the distribution of estimators $\hat{s}$, given true overlap *s* and colonies *c* is given by

$$P(\hat{s}|s,c)=\sum_{n_{a},n_{b},n_{ab}}\{P\left(\hat{s}|n_{a},n_{b},n_{ab}\right)\times P\left(n_{ab}|n_{a},n_{b},s\right)P\left(n_{b}|c\right)P\left(n_{a}|c\right)\}\;.$$ (11)

**Fig 6:**
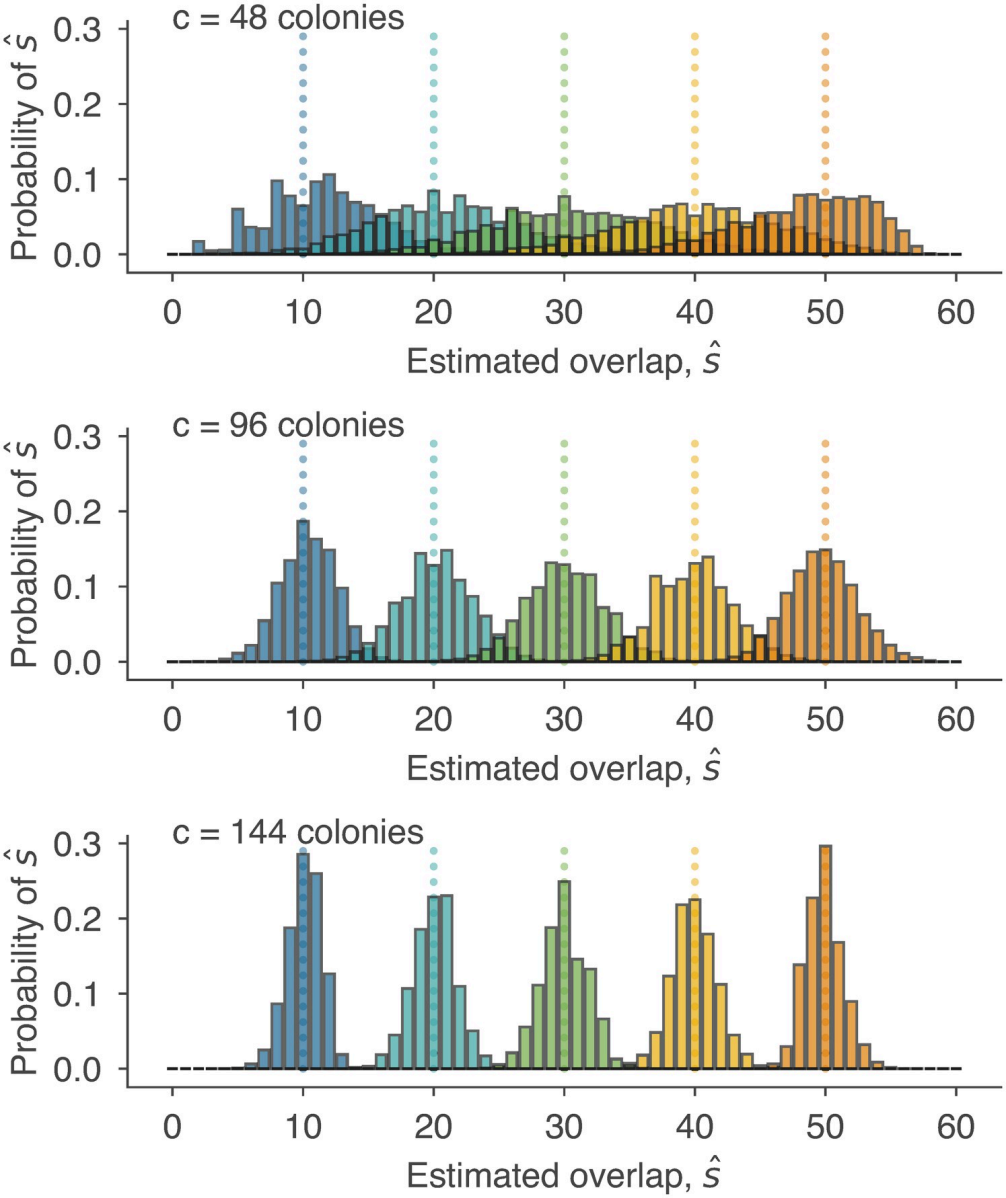
Quantifying the decrease in uncertainty from increased sequencing. Histograms show distributions of overlap estimates $\hat{s}$, computed using Eq (11), for various values of *s* which are indicated by color-matched dotted lines. While all estimates are distributed around the true values of *s*, increasing the number of colonies *c* from 48 (top) to 96 (middle) and to 144 (bottom) substantially decreases the error of estimates. For example the bottom plot shows that successfully sequencing *c* = 144 colonies from each parasite is guaranteed to produce estimates $\hat{s}$ that are off by at most 5 (8.3%) in either direction of the true *s*.

$P(\hat{s}|n_{a},n_{b},n_{ab})$ is the probability of getting a particular estimate $\hat{s}$, given information about coverage and overlap. In fact, this is a distribution concentrated at a single point, i.e., a Dirac *δ* function, since each triple (*n*_*a*_, *n*_*b*_, *n*_*ab*_) maps to exactly one point estimate $\hat{s}$. As a result, this term tells us the locations at which there will be probability mass, while the remaining terms in Eq (11) tell us how much mass there will be at those locations. In other words, this distribution is a discrete probability distribution, and we have written down a fancy form of it above. By aggregating into bins, this distribution can be conveniently visualized as a histogram, which shows how the uncertainty of estimators depends on the true overlap *s* and the sequencing effort *c*. Fig 6 shows the effect of increasing sequencing efforts from a half plate (*c* = 48) to a full 96-well plate (*c* = 96) and beyond. These calculations succinctly quantify intuition: additional laboratory efforts lead to higher accuracy guarantees.

The calculations and distributions in this section show how the Bayesian framework in this manuscript can also be used to plan sequencing studies and estimate study costs. If a desired downstream analysis of repertoire overlap requires results that are accurate to within a particular number of shared sequences, BRO methods can easily specify the sequencing efforts needed.

## Discussion

This manuscript places the estimation of overlap between fixed-size repertoires or populations from incomplete samples on firm statistical ground. While myriad indices of *β*-diversity for presence-absence data exist [2], they implicitly treat species counts as complete, leading to bias. Notable exceptions which embrace imperfect sampling exist [7], but require species abundance data to compute. Here, we clearly define a stochastic process for fixed-size repertoires that generates sample presence/absence data, opening the door to rigorous Bayesian inference. In particular, Eq (7) provides point estimates of true repertoire overlap, while Eq (8) provides error bars and uncertainty estimates via credible intervals. If desired, improved estimates of $\hat{s}$ can be plugged directly into any of the dozens of presence/absence measures of similarity reviewed in Ref. [2]. Figs 3 and 4 show the consistency and accuracy of these calculations across simulated sampling regimes in which the correct answer is known.

Bayesian repertoire overlap (BRO) is also useful in real-data scenarios, when the correct answer is unknown. By revisiting previously published studies of the *var* genes of *P*. *falciparum* [18, 19, 21, 27], we showed that switching from the Sørenson-Dice coefficient $\overset{˚}{S}$ (called pairwise type sharing in the malaria literature) to BRO leads to different conclusions (Fig 5 left, middle) or high uncertainty (Fig 5 right). In particular, these reanalyses point to a clear recommendation for the design of future malaria studies: the number of unique *var* sequences per isolate should be at least 30. Since each additional PCR product may not contribute an additional unique sequence, we again used the Bayesian framework to translate increased PCR efforts to decreased uncertainty (Fig 6). Accuracy requirements can now be weighed against laboratory costs during the planning of studies.

While BRO clearly outperforms $\overset{˚}{S}$ in practical contexts, it is also more cumbersome to compute. Indeed, $\overset{˚}{S}$ can be calculated on the back of an envelope while Eq (7) requires a computer, or at least a lot more envelopes. However, as it turns out, there are only around 77, 500 possible combinations of *n*_*a*_, *n*_*b*_, and *n*_*ab*_, which means that a lookup table of every conceivable $\hat{s}$ value can be computed on a laptop in minutes and attached to an email. Links to open-source code and a convenient web tool can be found in the Acknowledgements.

The models introduced in this paper are as correct as their assumptions, which we now revisit. During the construction of the Bayesian repertoire overlap, we assumed that our prior distribution *P*(*s*) was uniform, meaning that we treated each possible level of overlap as equally likely. This is easily defensible in practice, as any other choice would introduce unacceptable bias.

We also assumed, when computing the tradeoff between sequencing effort and uncertainty, that each sequence in each repertoire was just as likely to have been sampled, which may or may not be true, for two distinct reasons. First, due to the fact that sequences are obtained using degenerate primers, the effects of primer bias may cause some sequences to be amplified more often than others. Second, a single parasite genome might have multiple copies of the exact same *var* gene, or might have distinct *var* genes whose DBL*α* tags are nevertheless identical. This scenario is arguably more likely among South American genomes whose overall *var* diversity is lower. Experimentally, the probability that a sequence is observed will be scaled upward by its genomic multiplicity, but the scaling may be non-linear since PCR protocols include many rounds of amplification, magnifying the deviations from uniformity. Fully addressing either of these possibilities would require that we modify the probabilities in both the coupon collector’s problem and the repertoire subsampling processes, and then use Monte Carlo methods to numerically compute posterior distributions.

New sampling protocols for *var* genes, based on next-generation sequencing methods [25], may or may or may not meet the assumptions of the estimator presented here. To use the hypergeometric distribution, we require that, if an entire sampling protocol were to be technically replicated many times, that eventually each member of the repertoire would be observed with equal probability. In other words, while fluctuations in any particular set of observations are expected, with technical replication those fluctuations must eventually even out, approaching uniformity. Thus, the issues of primer bias and gene multiplicity violate the assumption of uniformity, but the magnification of random initial fluctuations, e.g., by the repeated amplification rounds of PCR, do not.

Could deviations from the modeling assumption of uniformity could be inferred from the data themselves? If so, this idea could in principle be applied to cloning-based methods and next-generation methods alike. This is an interesting direction for future work, and could draw from advances in abundance-based estimators for *β*-diversity [7, 12], or could incorporate explicit knowledge of the effects of protocols and pipelines, in order to mathematically undo their effects.

More practically, the assumption that the *var* repertoire size is 60 makes the methods of this paper less useful in the context of complex infections with multiple parasite genomes [23, 27]. In cases where the multiplicity of infection is known, overlap estimates could be computed using generalizations of the statistics in this paper, computing overlap between infections (instead of between parasites). This would be complicated by possible overlap of parasite repertoires within each infection, but repertoires tend to be quite different in areas of high transmission so the methods herein may be approximately correct. Nevertheless, development of more sophisticated methods would be especially useful in the context of *var*-based epidemiological studies.

This paper focuses on malaria’s *var* genes, and assumes a total repertoire size of 60, but mathematically relaxing this assumption (S1 Text) broadens the set of possible applications. First, within studies of malaria’s *var* genes, the total repertoire size fluctuates slightly from parasite to parasite. As larger whole-genome datasets become available, this information can be incorporated directly as a prior over the distribution of repertoire sizes, improving estimates further. This opens the door to the analysis of *Plasmodium spp*. multigene antigen families such as *rif* and *stevor* [32, 33], or more general studies of *β*-diversity in multigene families in which population sizes are fixed or their size distributions have been sampled [34]. Second, outside of malaria, improved estimators may also be useful in comparing, for instance, the genomic archives of antigen-encoding *vsg* genes used by *Trypanosoma brucei* for immune evasion [35]. Finally, the mathematics of this paper need not be applied to genetics or even within ecology; large-data applications like Facebook and other online social networks compute the sizes of intersections of sets—how many interests or friends do two individuals have in common?—but use only subsamples of data to decrease computation time in distributed computing settings [13].

Finally, this work presents a Bayesian approach to inference of *β*-diversity under particular assumptions, which contrasts the vast majority of indices, coefficients, and metrics to date which remain non-probabilistic [2]. Changing the underlying assumptions of the Bayesian repertoire overlap method, or the statistics of the sampling process, would lead to additional estimators for other common cases. Across applications, unbiased estimation combined with the quantification of uncertainty will allow for more reliable results and better prospective study design.

## Supporting information

S1 FigBayesian repertoire overlap consistently estimates true overlap for varying population size and sampling rates.Repertoires with true overlaps ranging from 0 to 60 were subsampled in simulations. While the main text shows results when *n*_*a*_ = *n*_*b*_ and when *N*_*a*_ = *N*_*b*_ = 60, these assumptions can also be relaxed. Increasing *N*_*b*_ from 60 (left column) to 120 (right column) does not affect the consistency of BRO estimates, nor does decreasing the number of samples from population *a* from *n*_*a*_ = 40 (top row) to *n*_*a*_ = 10 (bottom row). As in the main text, the underestimating bias of $\overset{˚}{S}$ is worse with lower sampling rates [7].(EPS)

S1 TextThe main text describes the Bayesian repertoire overlap estimator $\hat{s}$ when both populations are of size 60.A general estimator is derived for populations of arbitrary and possibly unequal size.(PDF)
