# Supplementary material for: Bayes-optimal estimation of overlap between populations of fixed size
Source: PLoS Comput Biol. 2019 Mar 29;15(3):e1006898. doi: 10.1371/journal.pcbi.1006898 (PMC6440621; doi:10.1371/journal.pcbi.1006898)
Supplement: S1 Text — A general estimator is derived for populations of arbitrary and possibly unequal size. (PDF) [file pcbi.1006898.s002.pdf]

S1 TEXT:
BAYES-OPTIMAL ESTIMATION OF OVERLAP BETWEEN POPULATIONS OF FIXED SIZE

How should one estimate the size of the intersection of two sets of arbitrary size from subsamples? As in the main text, assume that set a has total size N_a, and n_a objects are drawn from it uniformly at random. Similarly, assume that set b has total size N_b, and that n_b objects are drawn from it uniformly at random. Suppose that the number of objects found among the samples of sizes n_a and n_b is, as in the main text, n_{ab}.

Without loss of generality, assume that $N_a \leq N_b$. We make this assumption because the maximum value of the true overlap s is then N_a, since the two sets cannot have an intersection larger than the smaller of the two sets. The estimator $\hat{s}$ is given by

$$P(s \mid n_a, n_b, n_{ab}, N_a, N_b) = \frac{\sum_{s_a=0}^{N_a} P(n_{ab} \mid n_b, s_a, N_b) P(s_a \mid n_a, s, N_a)}{\sum_{s'=0}^{N_a} \sum_{s_a=0}^{N_a} P(n_{ab} \mid n_b, s_a, N_b) P(s_a \mid n_a, s', N_a)}, \tag{S1}$$

and

$$\hat{s} = \sum_{s=0}^{N_a} s \, P(s \mid n_a, n_b, n_{ab}, N_a, N_b), \tag{S2}$$

where we are now explicit about the total number of objects in the hypergeometric distributions—in the main text, these were implicitly 60. In other words, $P(x \mid t, u, v)$ is the hypergeometric probability of drawing exactly x special objects out of t draws, from a population of size v, in which there are u special objects total.

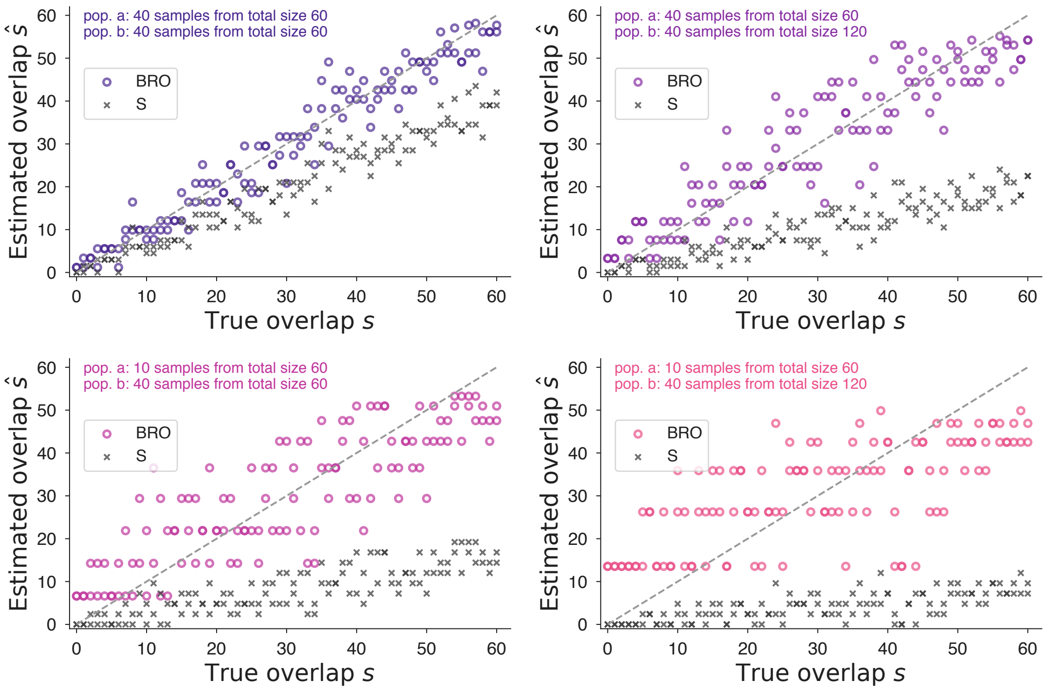

FIG. S1. **Bayesian repertoire overlap consistently estimates true overlap for varying population size and sampling rates.** Repertoires with true overlaps ranging from 0 to 60 were subsampled in simulations. While the main text shows results when $n_a = n_b$ and when $N_a = N_b = 60$, these assumptions can also be relaxed. Increasing N_b from 60 (left column) to 120 (right column) does not affect the consistency of BRO estimates, nor does decreasing the number of samples from population a from $n_a = 40$ (top row) to $n_a = 10$ (bottom row). As in the main text, the underestimating bias of $\mathring{S}$ is worse with lower sampling rates [7].
